# [^18^F]FDG PET/CT versus [^18^F]FDG PET/MRI in staging of non-small cell lung cancer: a head-to-head comparative meta-analysis

**DOI:** 10.3389/fmed.2024.1517805

**Published:** 2025-01-13

**Authors:** Dandan Yu, Chaolin Chen

**Affiliations:** Department of Clinical Pharmacy, Traditional Chinese Medical Hospital of Zhuji, Shaoxing, China

**Keywords:** [^18^F]FDG PET/CT, [^18^F]FDG PET/MRI, non-small cell lung cancer, meta-analaysis, staging

## Abstract

**Purpose:**

This meta-analysis aims to compare the diagnostic efficacy of [^18^F]FDG PET/CT and [^18^F]FDG PET/MRI in patients with non-small cell lung cancer (NSCLC).

**Methods:**

An extensive literature search was conducted throughout the PubMed, Embase, and Web of Science databases for works accessible through September 2024. We included studies assessed the diagnostic efficacy of [^18^F]FDG PET/CT and [^18^F]FDG PET/MRI in NSCLC.

**Results:**

The meta-analysis includes six studies with a total of 437 patients. The sensitivity and specificity of [^18^F]FDG PET/CT and [^18^F]FDG PET/MRI for detecting lymph node metastasis were similar, at 0.82 (0.68–0.94) vs. 0.86 (0.70–0.97) and 0.88 (0.76–0.96) vs. 0.90 (0.85–0.94), respectively, with no significant differences (*p* = 0.70 for sensitivity, *p* = 0.75 for specificity). For distant metastasis, the sensitivity of [^18^F]FDG PET/CT and [^18^F]FDG PET/MRI was 0.86 (0.60–1.00) and 0.93 (0.63–1.00), and specificity was 0.89 (0.65–1.00) vs. 0.90 (0.64–1.00), respectively, also showing no significant differences (*p* = 0.66 for sensitivity, *p* = 0.97 for specificity).

**Conclusion:**

Our meta-analysis shows that [^18^F]FDG PET/MRI has similar sensitivity and specificity to [^18^F]FDG PET/CT in identifying lymph node and distant metastases in patients with NSCLC. Additional larger sample prospective studies are needed to confirm these findings.

**Systematic review registration:**

https://www.crd.york.ac.uk/prospero/display_record.php?ID=CRD42023479817, CRD42023479817.

## 1 Introduction

Lung cancer is recognized as the most typical diagnosis malignancy globally, also notable for its high mortality rates (1). Lung cancer remains the most prevalent cancer globally in 2022, accounting for approximately 2.5 million new cases, which represents one in eight cancer diagnoses worldwide (12.4% of all global cancer incidences) (2). In this setting, roughly 80% of lung malignancies are categorized as non-small cell lung cancer (NSCLC), which is the main cancer diagnosis worldwide (3, 4). Surgery, radiation, chemotherapy, immunotherapy, and targeted therapy can all be used to treat NSCLC, depending on the stage of the tumor (5). The effectiveness of these treatments and the overall prognosis of the patient are profoundly impacted by the initial stage of the cancer (6). As a result, thorough and precise imaging-based staging is important for optimal care of NSCLC patients.

Currently, clinical methods used for NSCLC staging include computed tomography (CT), magnetic resonance imaging (MRI), and biopsy (7, 8). However, each of these modalities has its inherent limitations. While CT scanning excels in identifying the tumor’s location and determining lymph node enlargement, its limited ability to determine or exclude mediastinal metastasis imposes certain constraints on the accurate staging of lung cancer (9). MRI is often considered less effective than CT for detecting small cancer lesions, due to its sensitivity to cardiac and respiratory motion artifacts, extremely low T2 values, lung magnetic field heterogeneity, and the low proton density of lung parenchyma (10). Biopsies, although crucial for delivering definitive results, are associated with inherent risks and may not always be feasible. The most common complication encountered is pneumothorax, which occurs in 20–64% of all CT-guided biopsies (11, 12). Additionally, hemorrhage from the lung parenchyma stands as another notable complication, frequently resulting from the needle track crossing a pulmonary vessel (13).

Positron emission tomography (PET) plays a crucial role in diagnosing NSCLC, from initial detection to staging and monitoring tumor metastasis (14). Integrating PET with 18F-fluorodeoxyglucose ([^18^F]FDG) into PET/CT and PET/MRI systems has considerably revolutionized cancer imaging by integrating metabolic and anatomical information (15). [^18^F]FDG PET/CT plays an important role in managing NSCLC, notably in evaluating the nodal status and finding occult metastatic disease, where it outperforms the capabilities of CT scanning alone (16). The NCCN guidelines emphasize the importance of rapid access to PET/CT for accurate staging in NSCLC, highlighting its role in guiding management decisions and predicting prognosis across all stages of the disease, particularly in detecting metastases (17). One of its main benefits over traditional imaging approaches is its increased sensitivity for detecting extra-thoracic metastases (18, 19). Dahlsgaard-Wallenius et al. found that PET/MRI and PET/CT had comparable diagnostic capacities for N-staging in NSCLC (20). Combining the metabolic data from PET with the special characteristics of MRI—such as low radiation exposure and excellent soft tissue contrast—makes PET/MRI an advantageous test (21). In several studies, evidence suggested that PET/MRI may outperform PET/CT in detecting metastases within the pleura, brain, liver, and bone (22, 23). This is also in accordance with the results of a prospective single-center research of 330 exams, where PET/MRI found brain and liver metastases that were undetectable by PET/CT (24). Thus, the use of a hybrid PET/MRI in lung cancer patients may sometimes assist the detection of distant metastases, because NSCLC metastases are primarily situated in the brain, liver, and bone (25, 26). However, the included trials gave minimal data on extra-thoracic metastatic illness, making it unable to draw conclusions about the potential advantage of PET/MRI. Due to the relative novelty of PET/MRI and the limited availability of direct comparison studies, inconsistencies in the literature regarding their comparative efficacy warrant careful examination.

The goal of this meta-analysis is to comprehensively evaluate the diagnostic performance of [^18^F]FDG PET/MRI to [^18^F]FDG PET/CT in NSCLC through head-to-head comparison.

## 2 Methods

The meta-analysis followed the Preferred Reporting Items for Systematic Reviews and Meta-Analyses of Diagnostic Test Accuracy (PRISMA-DTA) standards (27). The protocol for this meta-analysis is registered with PROSPERO (CRD42023479817).

### 2.1 Search strategy

An extensive literature search was conducted in PubMed, Embase, and Web of Science to identify pertinent publications available up to September 2024. The search utilized the following keywords: (“PET/MRI” or “PET/CT”) AND (“lymph node metastasis”) AND (“distant metastasis”) AND (“non-small cell lung cancer”). Further details are available in Supplementary Table S1. The reference lists of the listed studies were meticulously manually examined to identify additional relevant literature.

### 2.2 Inclusion and exclusion criteria

This meta-analysis included studies that satisfied the PICOS framework: Population (P): patients diagnosed with NSCLC; Intervention (I): diagnostic imaging using [^18^F]FDG PET/CT and/or [^18^F]FDG PET/MRI; Comparison (C): studies comparing PET/CT and PET/MRI; Outcomes (O): studies that report diagnostic performance in assessing lymph node involvement and/or distant metastases; Study design (S): studies with a sample size greater than ten.

Studies were excluded if they were (1) animal studies, (2) non-research articles such as reviews, case reports, conference abstracts, meta-analyses, letters to the editor, or (3) non-randomized designs including case–control, cohort, and cross-sectional studies. Additionally, studies employing other radiotracers were also omitted. For studies utilizing the same data sets, only the most recent were considered.

### 2.3 Quality assessment

Two researchers independently evaluated the quality of the included studies using the Quality Assessment of Diagnostic Accuracy Studies-2 (QUADAS-2) tool (28). This tool addresses four key domains: patient selection, index test, reference standard, and flow and timing. Each study was independently rated, and any disagreements were resolved through discussion to reach consensus. The QUADAS-2 tool allowed for a structured and transparent appraisal of study quality, highlighting areas with potential risk of bias or applicability concerns.

### 2.4 Data extraction

Two researchers extracted data separately from the selected papers. This data encompassed details as author, year of publication, imaging test type, study characteristics (country, study design, study duration, analysis, and reference standard), patient characteristics (number of patients, radiologists involved, and mean/median age), and technical specifics [scanner modality, ligand dose, image analysis, and true positives (TP), false positives (FP), false negatives (FN), true negatives (TN)].

### 2.5 Outcome measures

The primary endpoints were the sensitivity and specificity of [^18^F]FDG PET/CT and [^18^F]FDG PET/MRI in detecting lymph node metastasis and distant metastasis. Sensitivity was defined as the proportion of TP scans relative to the sum of TP and FN scans, reported at either the patient or lesion level. Specificity was defined as the proportion of TN scans relative to the total of TN and FP scans, as documented.

### 2.6 Statistical analysis

The DerSimonian and Laird methods were used to assess sensitivity and specificity, which were then combined with the Freeman-Tukey double inverse sine transformation. Confidence intervals were calculated employing the Jackson method. Heterogeneity both within and across groups was evaluated using the Cochrane Q and I^2^ statistics (29). Significant heterogeneity (*p* < 0.10 or I^2^ > 50%) warranted sensitivity analysis and meta-regression to identify individual studies contributing to heterogeneity.

Both funnel plots and Egger’s test were used to investigate publication bias. For all statistical analyses, a significance level of *p* < 0.05 was set. R software version 4.1.2 was used for computation and graphical display of statistical analyses.

## 3 Results

### 3.1 Study selection

**Figure 1:**
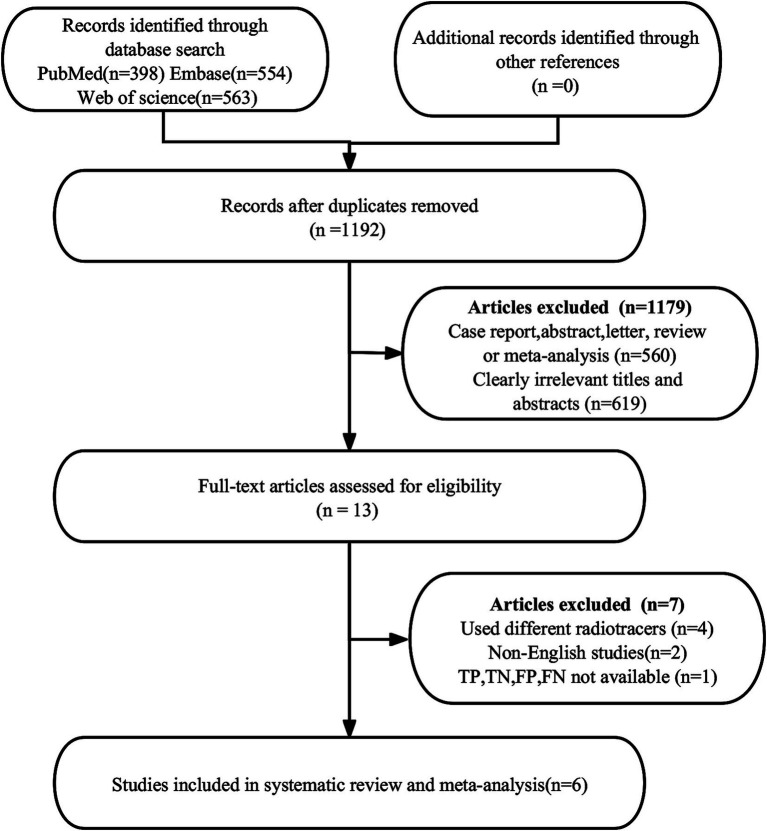
PRISMA flow diagram illustrating the study selection process.

A total of 1,515 publications were found in the first search. Nevertheless, 323 studies were found to be duplicates and were not eligible for this study, leaving 1,192 studies for further analysis. After a thorough review of the remaining 13 articles, 7 more were deemed ineligible due to unavailable data (TP, FP, FN, and TN) (*n* = 1) or different radiotracers (*n* = 4). Additionally, non-English articles (*n* = 2) were excluded. Ultimately, the meta-analysis included 6 articles (23, 30–34) evaluating the diagnostic efficacy of [^18^F]FDG PET/CT and [^18^F]FDG PET/MRI. The article PRISMA selection process is illustrated in Figure 1.

### 3.2 Study description and quality assessment

The six qualifying trials included a total of 437 NSCLC patients aged 35 to 89. All included articles were prospective design. All studies included N-stage evaluations, and three studies provided data regarding distant metastasis (23, 31, 32). Concerning analysis methods, each of the six articles employed patient-level analysis. Two articles adopted pathology as the reference standard, whereas four utilized either pathology or follow-up imaging for this purpose. Table 1 shows the study and patient characteristics for [^18^F]FDG PET/CT and [^18^F]FDG PET/MRI, whereas Tables 2, 3 describes the technical parameters.

**Table 1:** Study and patient characteristics of the included studies.

Author	Year	Country	Study duration	Study design	Analysis	Reference standard	No. of expert readers	No. of patients	Mean/median age
Heusch et al.	2014	Germany	NA	Pro	PB	Pathology	2	22	Mean ± SD:65.1 ± 9.1
Ohno et al.	2015	Japan	2012–2013	Pro	PB	Pathology	2	140	Mean ± SD:72.0 ± 7.4
Huellner et al.	2016	Switzerland	2012–2014	Pro	PB	Pathology and/or follow-up imaging	2	42	Median(range):65(35–89)
Lee et al.	2016	Korea	2013–2014	Pro	PB	Pathology and/or follow-up imaging	3	45	Mean ± SD:62.9 ± 9.9
Kirchner et al.	2018	Germany	NA	Pro	PB	Pathology and/or follow-up imaging	2	84	Mean ± SD:62.5 ± 9.1
Ohno et al.	2020	Japan	2014–2015	Pro	PB	Pathology and/or follow-up imaging	2	104	Mean ± SD:71.1 ± 6.3

PB patient-based; LB lesion-based; pro prospective; retro retrospective; NA not available.

**Table 2:** Technical aspects of included studies.

Author	Year, journal	Histological subtypes (percentage)	Distribution of TNM stages (percentage)	Manufacturer for PET/CT	Manufacturer and magnet strength for PET/MRI	Ligand dose	Image analysis
Heusch et al.	2014, Journal of Nuclear Medicine	Adenocarcinoma: 63.6%, Squamous cell carcinoma: 22.7%, Large cell carcinoma: 13.6%	NA	Siemens Molecular Imaging	Siemens Healthcare, Biograph mMR, 1.5 T	300 ± 45 MBq	Visual and semiquantitative
Ohno et al.	2015, Radiology	Adenocarcinoma:87.9%, Squamous cell carcinoma:9.3%, Adenosquamous cell carcinoma:2.1%, Large cell carcinoma: 0.7%	T stages: T1a 14.3%, T1b 37.1%, T2a 21.4%, T2b 14.3%, T3 7.1%, T4 5.7%; N stages: N0 55.7%, N1 24.3%, N2 11.4%, N3 8.6%; M stages: M0 88.6%, M1a 4.3%, M1b 7.1%	Aquilion 64 and One, Toshiba Medical Systems	GE Healthcare, Signa Excite XL Echospeed, 1.5 T; Philips Healthcare, Achieva 1.5 T	3.3 MBq/kg	Visual
Huellner et al.	2016, Journal of Nuclear Medicine	NA	NA	Discovery PET/CT 690 VCT; GE Healthcare	GE Healthcare, Discovery MR 750w, 1.5 T	350 MBq	Visual and semiquantitative
Lee et al.	2016, European Radiology	Adenocarcinoma: 71.1%, Squamous cell carcinoma: 17.8%, Other subtypes: 11.1%	T stages: T1 32.5%, T2 52.5%, T3 15.0%; N stages: N0 50.0%, N1 16.7%, N2 28.6%, N3 4.8%; M stages: M0 86.7%, M1 13.3%	Siemens Medical Solutions, Knoxville, TN	Siemens Healthcare, Biograph mMR, 1.5 T	5.2 MBq/kg	Visual and semiquantitative
Kirchner et al.	2018, European Journal of Nuclear Medicine and Molecular Imaging	Adenocarcinoma: 70.2%, Squamous cell carcinoma: 25.0%, Large cell carcinoma: 2.4%, Others: 2.4%	NA	Siemens Healthcare GmbH, Erlangen, Germany	Siemens Healthcare GmbH, Biograph mMR, 1.5 T	275.7 ± 47.4 MBq	Visual and semiquantitative
Ohno et al.	2020, American Journal of Roentgenology	Adenocarcinoma: 74%; Squamous cell carcinoma: 20.2%, Large cell carcinoma: 5.8%	T stages: T1 35.6%, T2 36.5%, T3 6.7%, T4 6.7%; N stages: N0 60.6%, N1 15.4%, N2 13.5%, N3 10.6%; M stages: M0 87.5%, M1 12.5%	Discovery ST Elite Performance, GE Healthcare	Canon Medical Systems, Vantage Titan 3 T	3.3 MBq/kg	Visual

NA not available; T primary tumor; N lymph node metastasis; M distant metastasis.

**Table 3:** Summary of 2×2 contingency table for diagnostic performance for N and M staging using [^18^F]FDG PET/CT and [^18^F]FDG PET/MRI.

Author	Modality	N staging				M staging				Total patients
		TP (No. patients)	FP (no. patients)	FN (no. patients)	TN (no. patients)	TP (no. patients)	FP (no. patients)	FN (no. patients)	TN (no. patients)	
Heusch et al. (30)	[^18^F]FDG PET/CT	6	2	2	12	NA	NA	NA	NA	22
	[^18^F]FDG PET/MRI	7	1	1	13	NA	NA	NA	NA	22
Ohno et al. (31)	[^18^F]FDG PET/CT	105	4	7	24	115	4	9	12	140
	[^18^F]FDG PET/MRI	112	2	0	36	124	2	0	14	140
Huellner et al. (32)	[^18^F]FDG PET/CT	14	3	1	24	10	3	5	24	42
	[^18^F]FDG PET/MRI	11	6	1	24	9	9	3	21	42
Lee et al. (23)	[^18^F]FDG PET/CT	10	14	6	12	8	11	7	16	42
	[^18^F]FDG PET/MRI	3	0	3	39	5	0	1	39	45
Kirchner et al. (33)	[^18^F]FDG PET/CT	42	1	5	36	NA	NA	NA	NA	84
	[^18^F]FDG PET/MRI	42	2	5	35	NA	NA	NA	NA	84
Ohno et al. (34)	[^18^F]FDG PET/CT	23	3	18	60	NA	NA	NA	NA	104
	[^18^F]FDG PET/MRI	33	8	8	55	NA	NA	NA	NA	104

TP true positive; TN true negative; FP false positive; FN false positive; N lymph node metastasis; M distant metastasis; NA not available.

**Figure 2:**
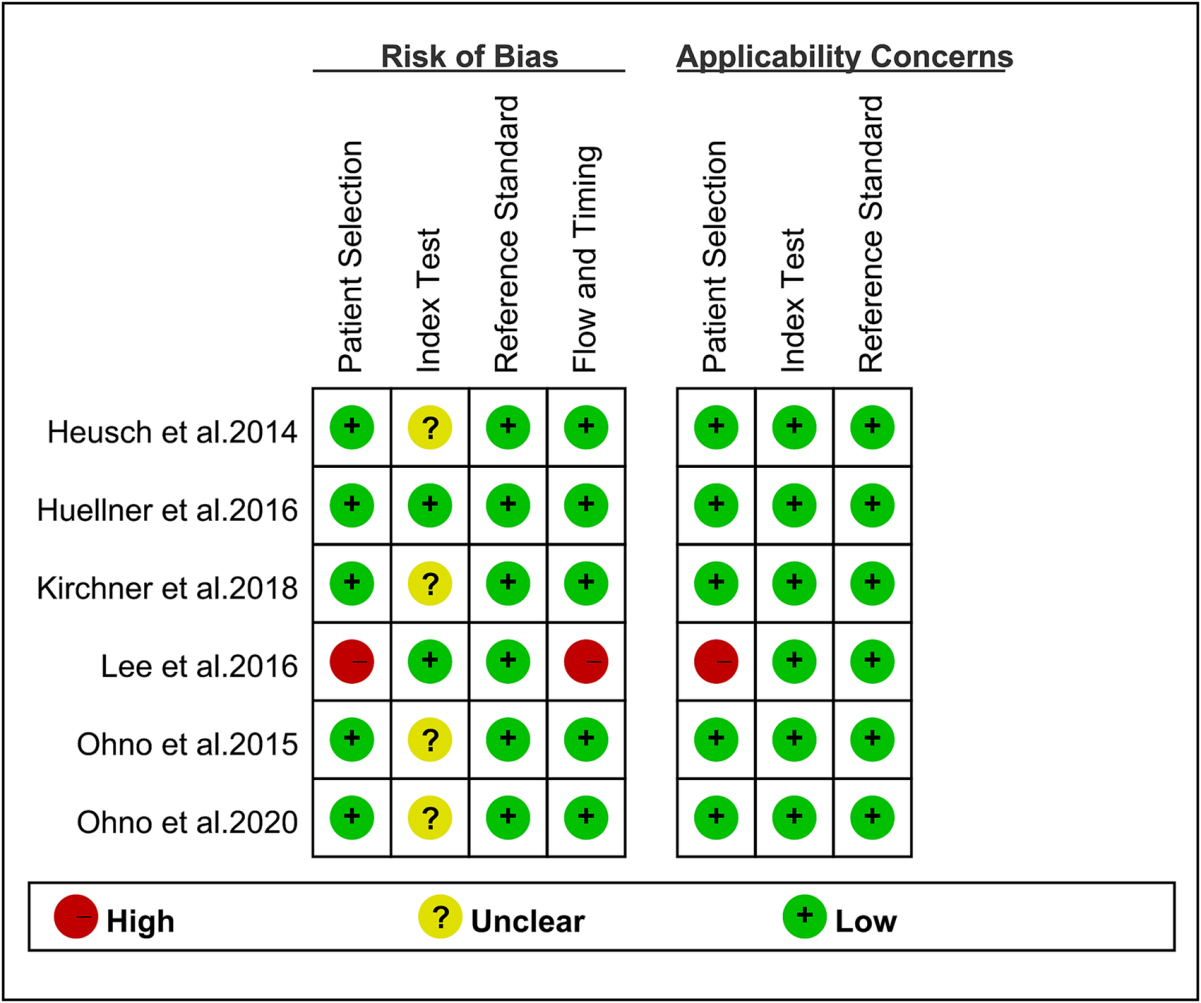
Risk of bias and applicability concerns of the included studies using the quality assessment of diagnostic performance studies QUADAS-2 tool.

Figure 2 depicts the risk of bias in each study, as assessed using the QUADAS-2 technique. When examining the risk of bias for patient selection, we discovered that one research was classified as “high risk” due to the absence of consecutive patients. One research classified the flow and timing criteria as “high risk” because certain subjects were excluded from the data analysis. The overall quality evaluation found that the included studies were good in quality.

### 3.3 Comparing the sensitivity of [^18^F]FDG PET/CT and [^18^F]FDG PET/MRI for detecting lymph node metastasis in NSCLC

**Figure 3:**
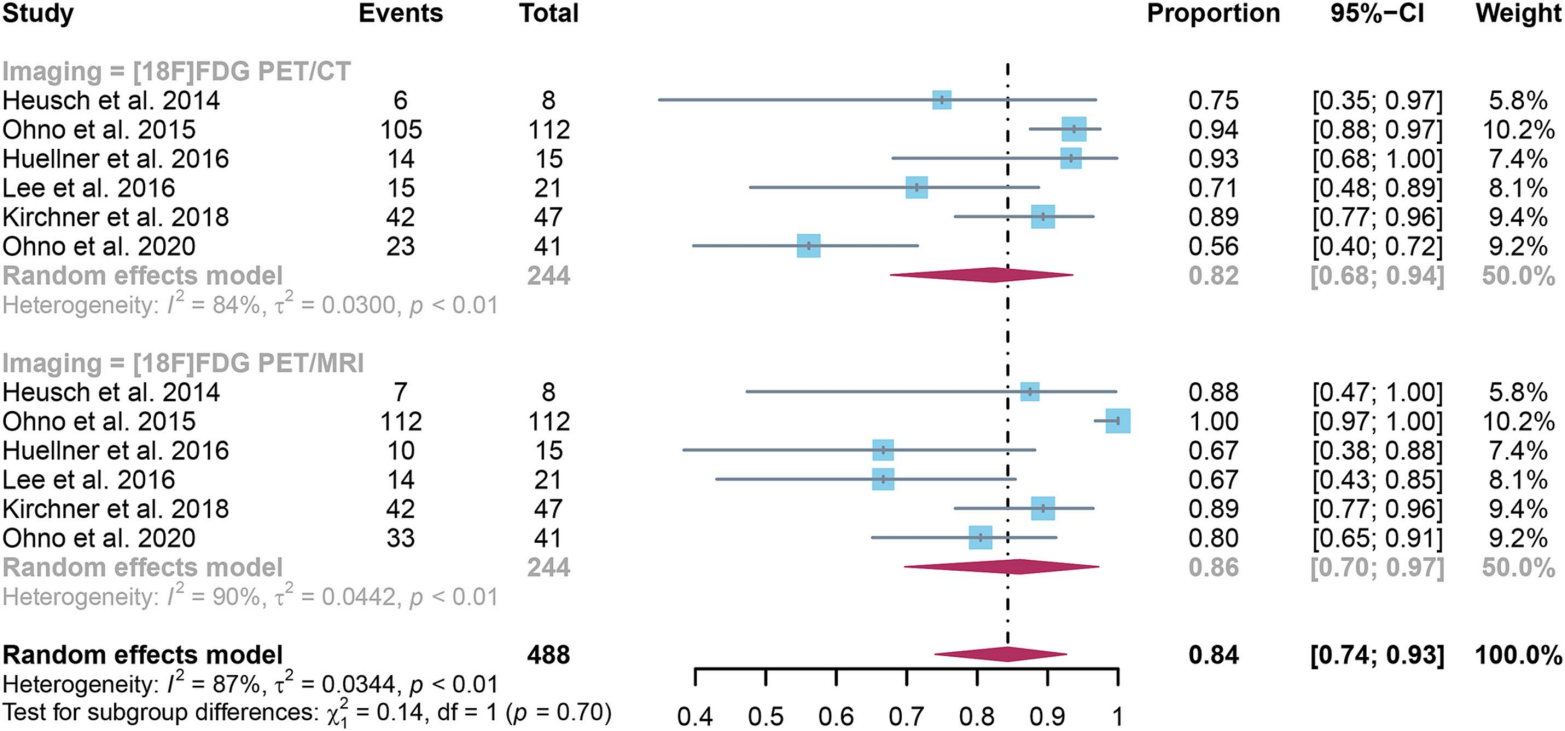
Forest plot of sensitivity comparison between [^18^F]FDG PET/CT and [^18^F]FDG PET/MRI in detecting lymph node metastasis in non-small cell lung cancer.

The analysis incorporated six studies, revealing a pooled sensitivity of 0.82 (95% CI: 0.68–0.94) for [^18^F]FDG PET/CT in detecting lymph node metastases in NSCLC. On the other hand, [^18^F]FDG PET/MRI showed an overall sensitivity of 0.86 (95% CI: 0.70–0.97). As shown in Figure 3, there was no discernible change in sensitivity between [^18^F]FDG PET/CT and [^18^F]FDG PET/MRI (*p* = 0.70).

I^2^ values for [^18^F]FDG PET/CT and [^18^F]FDG PET/MRI were 84 and 90%, respectively. No discernible sources of heterogeneity were found using leave-one-out sensitivity analysis (Supplementary Figures S1, S2). The meta-regression analysis for [^18^F]FDG PET/CT also failed to find the origin of heterogeneity (Table 4). According to the meta-regression analysis for [^18^F]FDG PET/MRI, the difference in reference standard (*p* = 0.01) might be the source of heterogeneity (Table 5).

**Table 4:** Subgroup and meta-regression analysis of lymph node metastasis detection for [^18^F]FDG PET/CT.

Covariate	Studies, *n*	Sensitivity (95%CI)	*p*-value	Specificity (95%CI)	*P*-value
Reference standard			0.49		0.78
Pathology	2	0.90[0.66–1.00]		0.86[0.73–0.95]	
Pathology and/or follow-up imaging	4	0.79[0.60–0.93]		0.88[0.69–0.99]	
Race			0.55		0.46
White	3	0.90(0.81–0.97)		0.93[0.83–0.99]	
Yellow	3	0.77(0.49–0.96)		0.83[0.57–0.99]	
Image analysis			0.83		0.55
Visual and semiquantitative	4	0.86(0.78–0.93)		0.88(0.80–0.94)	
Visual	2	0.86(0.80–0.92)		0.93(0.86–0.98)	

**Table 5:** Subgroup and meta-regression analysis of lymph node metastasis detection for [^18^F]FDG PET/MRI.

Covariate	Studies, *n*	Sensitivity (95%CI)	*P*-value
Reference standard			0.01
Pathology	2	0.99[0.74–1.00]	
Pathology and/or follow-up imaging	4	0.79[0.66–0.89]	
Race			0.70
White	3	0.84[0.67–0.96]	
Yellow	3	0.88[0.58–1.00]	
Image analysis			0.22
Visual and semiquantitative	4	0.82[0.73–0.90]	
Visual	2	0.98[0.95–1.00]	

### 3.4 Comparing the specificity of [^18^F]FDG PET/CT and [^18^F]FDG PET/MRI for detecting lymph node metastases in NSCLC

**Figure 4:**
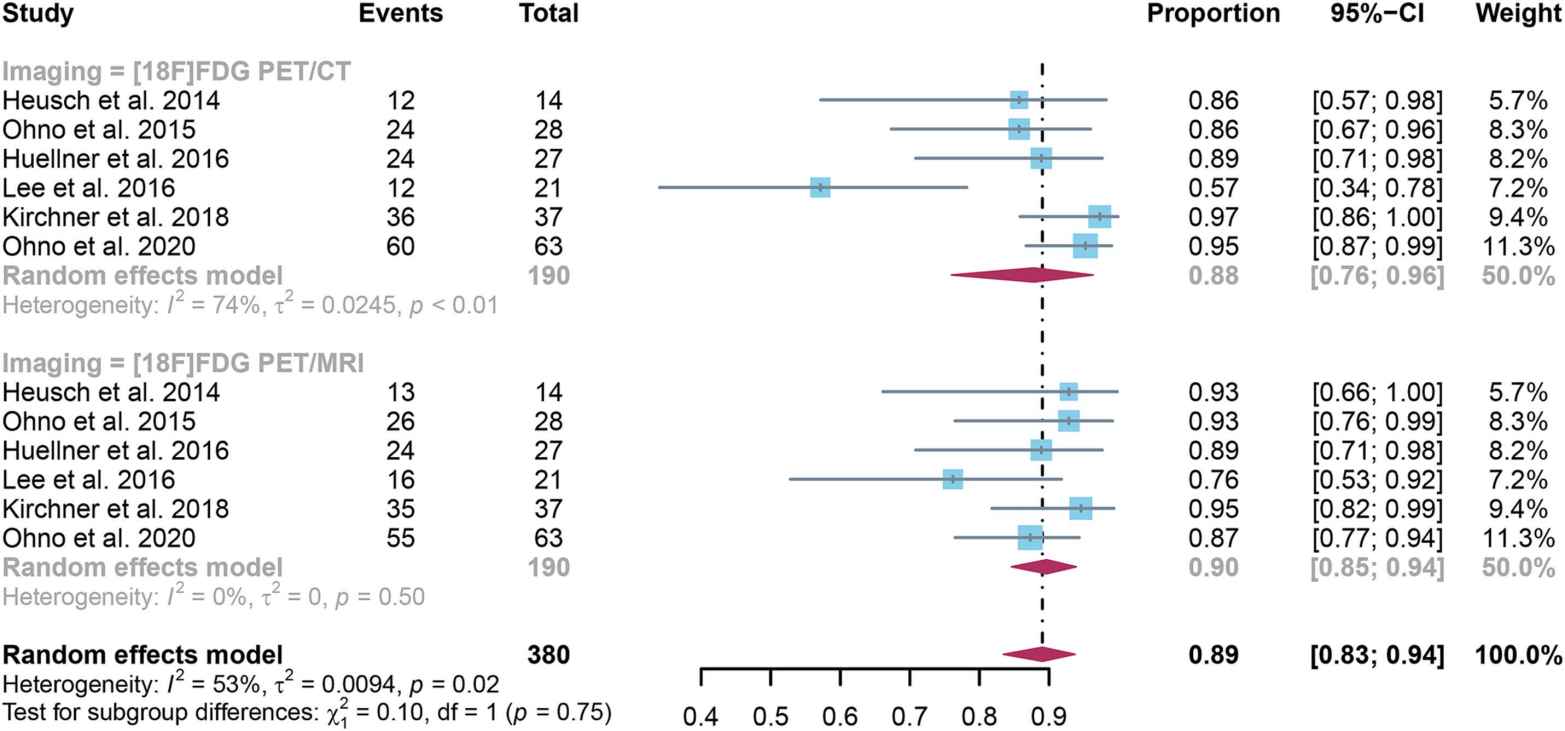
Forest plot of specificity comparison between [^18^F]FDG PET/CT and [^18^F]FDG PET/MRI in detecting lymph node metastasis in non-small cell lung cancer.

Six studies were included, and a pooled specificity of 0.88 (95% CI: 0.76–0.96) for [^18^F]FDG PET/CT in identifying lymph node metastases in NSCLC. In contrast, [^18^F]FDG PET/MRI had a pooled specificity of 0.90 (95% CI: 0.85–0.94) (Figure 4). There was no significant difference in specificity between [^18^F]FDG PET/CT and [^18^F]FDG PET/MRI (*p* = 0.75).

The I^2^ for sensitivity of [^18^F]FDG PET/CT was 74%. After omitting Lee et al.’s study, the I^2^ value reduced to 21%, indicating that this study might be a source of heterogeneity. Nonetheless, the findings of the specificity study were similar, with only modest differences between 0.85 and 0.93, as shown in Supplementary Figure S3.

### 3.5 Comparing the sensitivity of [^18^F]FDG PET/CT and [^18^F]FDG PET/MRI for detecting distant metastases in NSCLC

**Figure 5:**
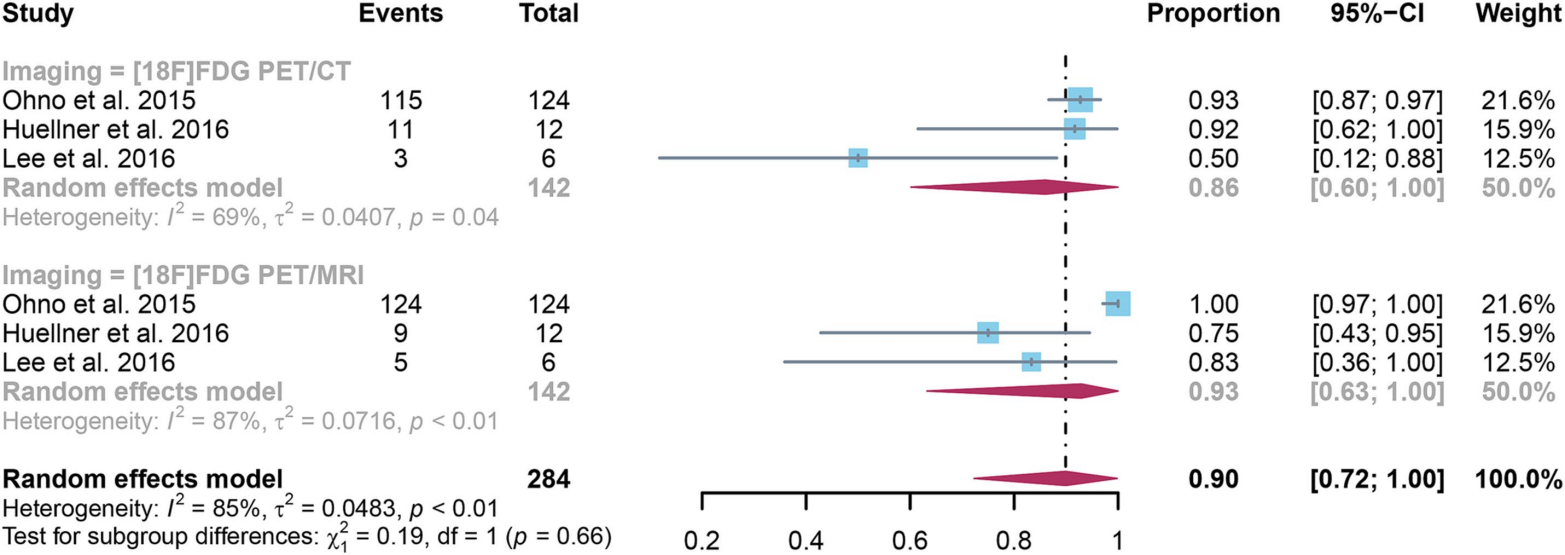
Forest plot of sensitivity comparison between [^18^F]FDG PET/CT and [^18^F]FDG PET/MRI in detecting distant metastasis in non-small cell lung cancer.

The analysis incorporated three studies, revealing a pooled sensitivity of 0.86 (95% CI: 0.60–1.00) for [^18^F]FDG PET/CT in detecting distant metastases in NSCLC. In contrast, [^18^F]FDG PET/MRI had an overall sensitivity of 0.93 (95% CI: 0.63–1.00) (Figure 5). There was no significant difference in sensitivity between [^18^F]FDG PET/CT and [^18^F]FDG PET/MRI (*p* = 0.66).

### 3.6 Comparing the specificity of [^18^F]FDG PET/CT and [^18^F]FDG PET/MRI for detecting distant metastases in NSCLC

**Figure 6:**
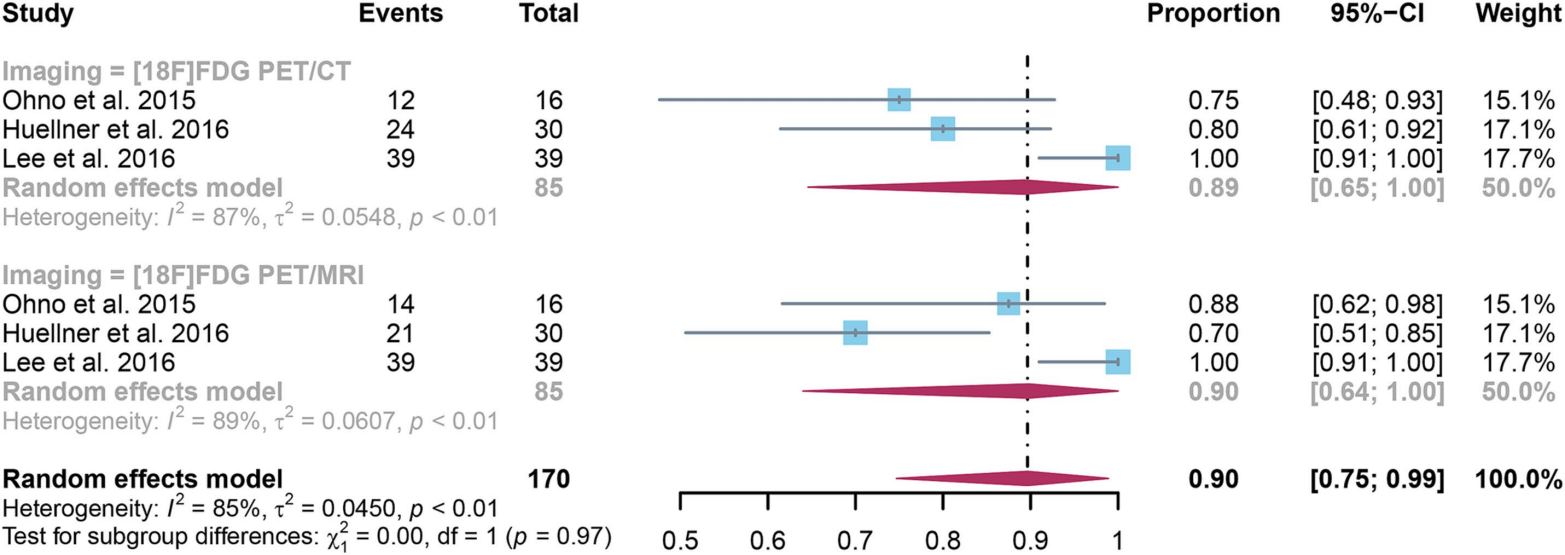
Forest plot of specificity comparison between [^18^F]FDG PET/CT and [^18^F]FDG PET/MRI in detecting distant metastasis in non-small cell lung cancer.

The analysis incorporated three studies, revealing a pooled specificity of 0.89 (95% CI: 0.65–1.00) for [^18^F]FDG PET/CT in detecting distant metastases in NSCLC. In contrast, [^18^F]FDG PET/MRI had an overall specificity of 0.90 (95% CI: 0.64–1.00) (Figure 6). There was no significant difference in specificity between [^18^F]FDG PET/CT and [^18^F]FDG PET/MRI (*p* = 0.97).

### 3.7 Publication bias

Funnel plot asymmetry tests were conducted to assess publication bias in [^18^F]FDG PET/CT and PET/MRI. For PET/CT, results indicated no significant bias for sensitivity (Egger’s *p* = 0.33, Supplementary Figure S4) or specificity (Egger’s *p* = 0.13, Supplementary Figure S5). For PET/MRI, significant bias was found in sensitivity (Egge’s *p* = 0.04, Supplementary Figure S6), while specificity showed no substantial bias (Egger’s *p* = 0.84, Supplementary Figure S7).

## 4 Discussion

The continuing controversy in the field of nuclear medicine regarding the comparative usefulness of [^18^F]FDG PET/CT and [^18^F]FDG PET/MRI in the assessment of lymph node and distant metastases in NSCLC requires a comprehensive meta-analysis (20, 22, 23). This analysis is critical for elucidating the diagnostic accuracy of these modalities, thereby informing clinical decision-making.

Our meta-analysis incorporated six studies to compare these imaging techniques. We discovered that [^18^F]FDG PET/CT had a pooled sensitivity of 0.82 and specificity of 0.88 in identifying lymph node metastases, whereas [^18^F]FDG PET/MRI had a sensitivity of 0.86 and specificity of 0.90, with no significant differences identified. Similarly, in identifying distant metastases, [^18^F]FDG PET/CT had a sensitivity of 0.86 and specificity of 0.89, whereas [^18^F]FDG PET/MRI had a sensitivity of 0.93 and specificity of 0.90, with no significant differences found. The slightly higher sensitivity of PET/MRI may be attributed to its superior soft tissue contrast provided by MRI, which enables better differentiation between tissues, especially in complex anatomical areas such as the lungs and lymph nodes (35). Unlike PET/CT, which uses X-ray imaging, MRI offers much higher resolution for soft tissue, allowing for more accurate detection of small or ambiguous lesions (36). However, the overlapping confidence intervals suggest that these differences might not be clinically significant.

Compared to the previous studies by Mojahed et al. (37) and Zhang et al. (35), which evaluated the diagnostic accuracy of [^18^F]FDG PET/CT versus [^18^F]FDG PET/MRI in T and N staging, our analysis reveals equivalent effectiveness of these modalities in detecting N and M stages in NSCLC patients. However, unlike Mojahed et al. and Zhang et al., we included evaluations of distant metastases, a crucial aspect of NSCLC staging. In addition to building on the previous analyses, our meta-analysis incorporates four new studies (23, 31, 32, 34), particularly those focusing on M stage (distant metastasis) assessment (23, 31, 32). This addition provides a more comprehensive understanding of [18F]FDG PET/MRI’s capabilities, addressing both nodal and metastatic assessments in NSCLC staging.

Zhang et al.’s (21) meta-analysis included 14 papers, five of which focused on lung cancer. In an analysis of five lung cancer trials including 429 patients, [^18^F]FDG PET/CT exhibited better sensitivity (0.87 vs. 0.84) and slightly worse specificity (0.95 vs. 0.96) than PET/MRI. In contrast, our meta-analysis found that [^18^F]FDG PET/MRI had similar sensitivity and specificity to [^18^F]FDG PET/CT in detecting lymph nodes and distant metastases in NSCLC patients. The discrepancy may stem from several factors. One key reason could be that Zhang et al.’s meta-analysis included patients with small cell lung cancer in addition to those with NSCLC. Small cell lung cancer generally presents with different patterns of lymph node and metastatic involvement compared to NSCLC, which may affect the diagnostic performance of [^18^F]FDG PET/CT and PET/MRI.

While PET/CT and PET/MRI modalities offer similar diagnostic efficacy, their cost and accessibility differ markedly, influencing their clinical integration. PET/CT, significantly more affordable, emerges as a cost-effective solution for healthcare providers (38). Its broader availability enhances its utility across diverse medical environments, proving especially advantageous in areas lacking advanced medical infrastructure. In instances where both techniques yield comparable sensitivity and specificity, PET/CT is frequently the preferred option. This preference stems not only from its cost-efficiency and wider accessibility, which promote extensive use, but also from its role in fostering more equitable healthcare access, particularly in under-resourced regions. Thus, balancing sophisticated diagnostic capabilities for practicalities such as affordability and accessibility, PET/CT distinctly outperforms when both modalities present equivalent diagnostic outcomes (36). A comprehensive comparison of the pros and cons of both imaging modalities is detailed in Supplementary Table S2.

Our study has some limitations. The inclusion of only six studies, and our analysis limited by the lack of detailed diagnostic performance data for different organ sites of metastasis, suggests a need for more extensive research in this area. Additionally, not all patients underwent pathological biopsy, some diagnoses were based on a combination of biopsy and clinical imaging follow-up. Future research should focus on studies using pathology as the sole gold standard to further validate these findings.

## 5 Conclusion

Our meta-analysis shows that [^18^F]FDG PET/MRI has similar sensitivity and specificity to [^18^F]FDG PET/CT in identifying lymph node and distant metastases in patients with NSCLC. Additional larger sample prospective studies are needed to confirm these findings.

## Data Availability

The original contributions presented in the study are included in the article/Supplementary material, further inquiries can be directed to the corresponding author.
