# Supplementary material for: [18F]FDG PET/CT versus [18F]FDG PET/MRI in staging of non-small cell lung cancer: a head-to-head comparative meta-analysis
Source: Front Med (Lausanne). 2025 Jan 13;11:1517805. doi: 10.3389/fmed.2024.1517805 (PMC11769939; doi:10.3389/fmed.2024.1517805)

Supplementary Table 1 Search strategy in PubMed, Embase and Web of Science.

| Database | Search strategy |
| --- | --- |
| PubMed | ("Carcinoma, Non-Small-Cell Lung"[Mesh] OR "Non-Small Cell Lung Carcinoma" [Title/Abstract] OR "Non-Small Cell Lung Cancer"[Title/Abstract] OR "NSCLC"[Title/Abstract]) AND ("lymphatic metastasis"[Mesh] OR "lymph node metastasis"[Title/Abstract] OR "nodal metastases"[Title/Abstract] OR “LNM” [Title/Abstract] OR “distant metastasis” [Title/Abstract]) AND ("Positron Emission Tomography Computed Tomography"[Mesh] OR "PET/MRI"[Title/Abstract] OR "PET/CT"[Title/Abstract] OR "positron emission tomography MRI"[Title/Abstract] OR "positron emission tomography CT"[Title/Abstract] OR "positron emission tomography Magnetic Resonance Imaging"[Title/Abstract] OR "positron emission tomography Computer Tomography"[Title/Abstract]) |
| Embase | ('non-small cell lung cancer'/exp) AND ('positron emission tomography-computed tomography'/exp) AND (‘lymph node metastasis’/exp OR ‘distant metastasis’:ab,ti) |
| Web of Science | ((TS=("Carcinoma, Non-Small-Cell Lung" OR "Non-Small Cell Lung Carcinoma" OR "Non-Small Cell Lung Cancer" OR "NSCLC")) AND TS=("lymphatic metastasis" OR "lymph nodes" OR "nodal metastases" OR “LNM” OR “distant metastasis”)) AND TS=("PET/MRI" OR "PET/CT" OR "positron emission tomography MRI" OR "positron emission tomography CT" OR "positron emission tomography Magnetic Resonance Imaging" OR "positron emission tomography Computer Tomography”) |

Supplementary Table 2 Pros and Cons of [18F]FDG PET/CT versus [18F]FDG PET/MRI.

| Criteria | FDG PET/CT | FDG PET/MRI |
| --- | --- | --- |
| Advantages | High spatial resolution for anatomic details due to CT. | Superior soft tissue contrast with MRI. |
| Resolution | High spatial resolution for anatomic details due to CT. | Superior soft tissue contrast with MRI. |
| Speed | Fast imaging process, widely available. | Longer scan time, but increasingly available. |
| Radiation Exposure | Ionizing radiation used for CT. | No ionizing radiation, MRI is safer for repeated use. |
| Cost | Relatively cost-effective compared to PET/MRI. | More expensive due to MRI system and longer imaging time. |
| Clinical Use | Standard of care for most cancers, widely accepted. | Growing use, especially in specific cases like brain and pelvic cancers. |
| Disadvantages | Inferior contrast for soft tissue differentiation. | Higher soft tissue contrast, ideal for complex anatomical areas. |
| Soft Tissue Contrast | Inferior contrast for soft tissue differentiation. | Higher soft tissue contrast, ideal for complex anatomical areas. |
| Bone Detail | Excellent bone detail, useful in skeletal metastasis. | Bone imaging less clear compared to CT. |
| Artifacts | Potential for artifacts due to motion or patient size. | Susceptible to motion artifacts and magnetic field interference. |
| Availability | More widely available, easier to integrate in routine practice. | Less available, requires advanced MRI infrastructure. |

Supplementary Figure 1 Sensitivity Analysis Assessing Studies on Sensitivity of [18F]FDG PET/CT for Lymph Node Metastasis in Non-Small Cell Lung Cancer.


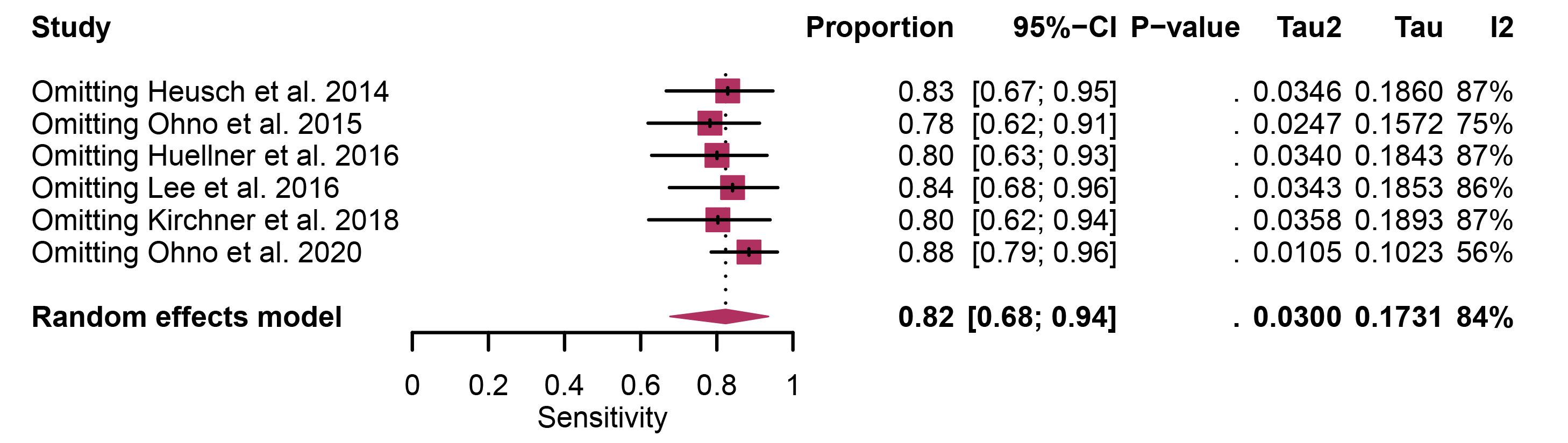


Supplementary Figure 2 Sensitivity Analysis Assessing Studies on Sensitivity of [18F]FDG PET/MRI for Lymph Node Metastasis in Non-Small Cell Lung Cancer.


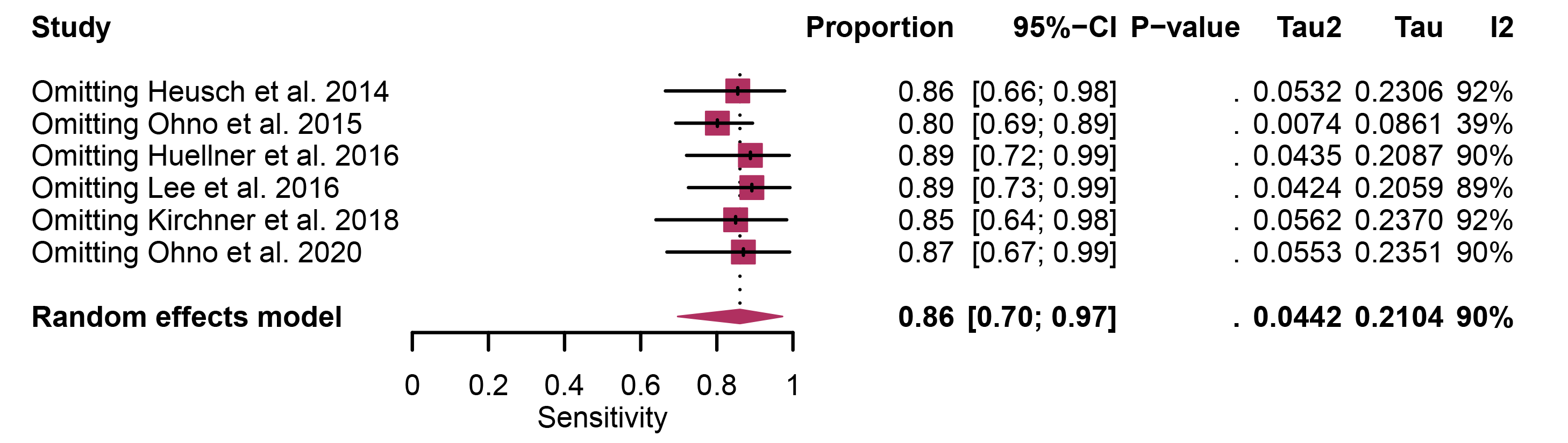


Supplementary Figure 3 Sensitivity Analysis Assessing Studies on Specificity of [18F]FDG PET/CT for Lymph Node Metastasis in Non-Small Cell Lung Cancer.


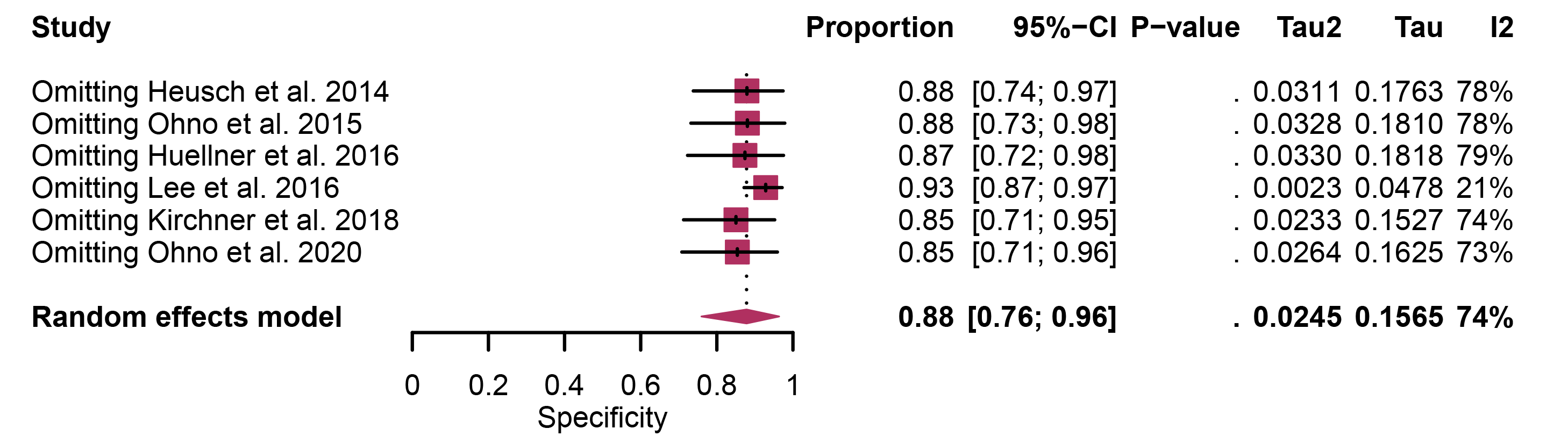


Supplementary Figure 4 Funnel Plot Assessing Publication Bias in Studies on Sensitivity of [18F]FDG PET/CT for Lymph Node Metastasis in Non-Small Cell Lung Cancer.


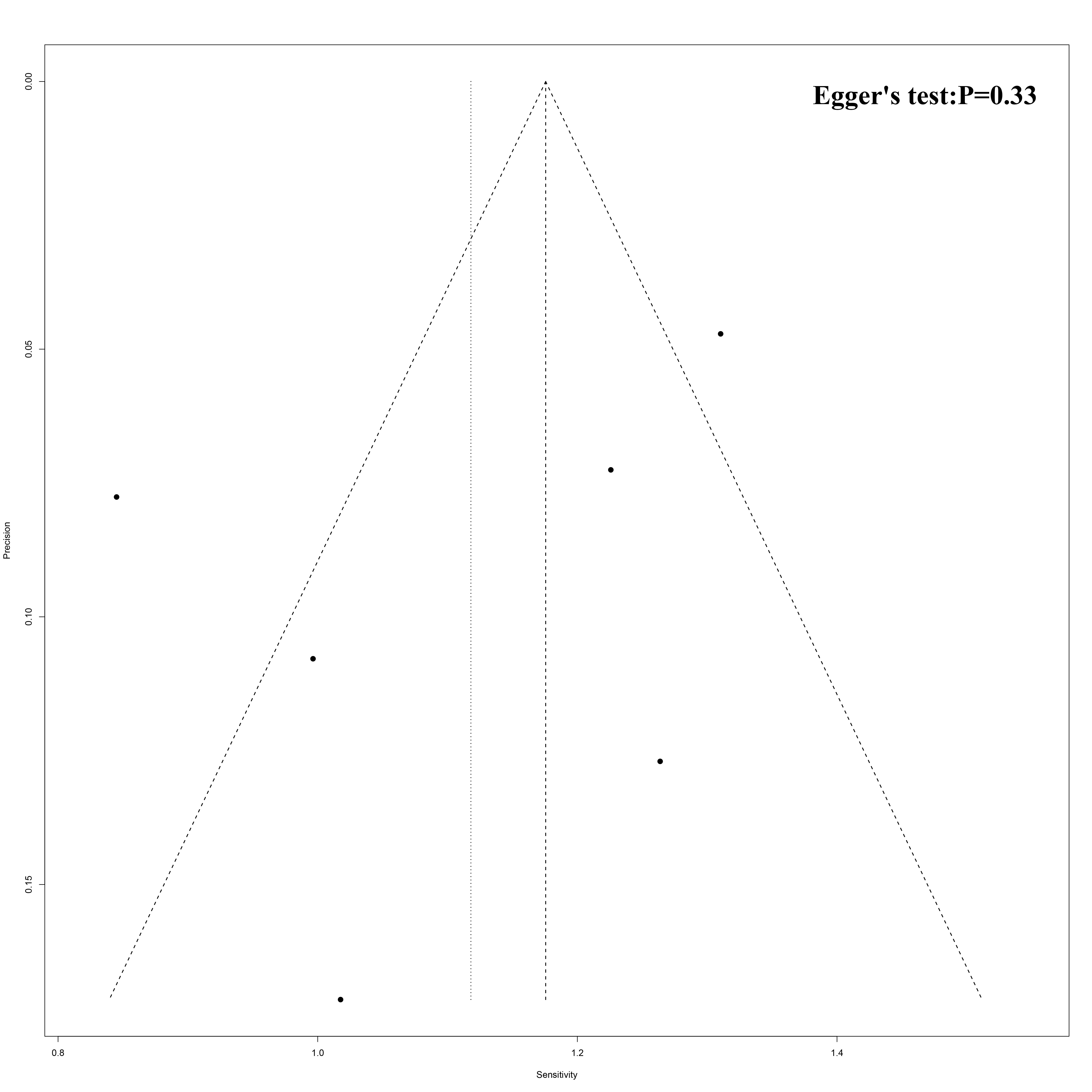


Supplementary Figure 5 Funnel Plot Assessing Publication Bias in Studies on Specificity of [18F]FDG PET/CT for Lymph Node Metastasis in Non-Small Cell Lung Cancer.


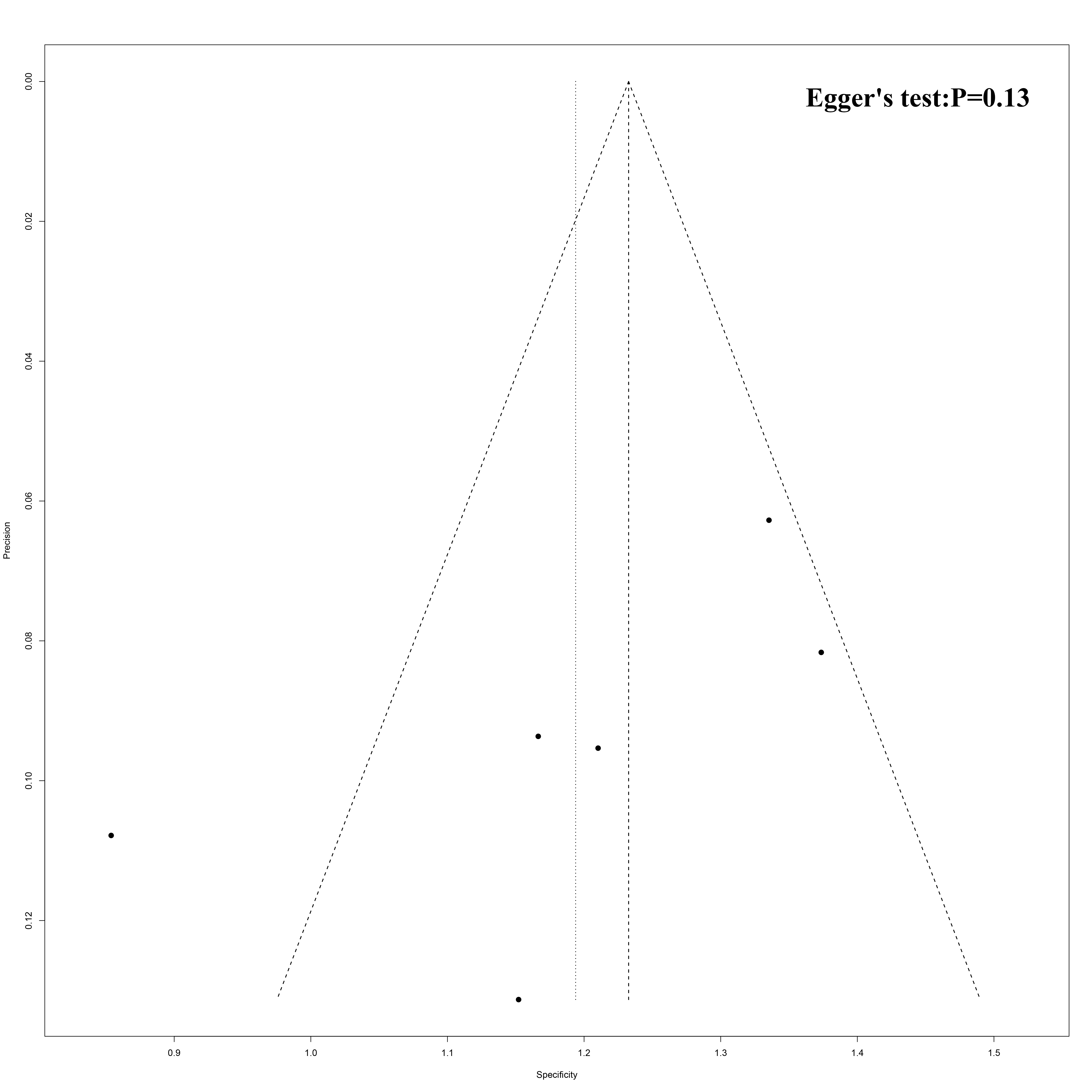


Supplementary Figure 6 Funnel Plot Assessing Publication Bias in Studies on Sensitivity of [18F]FDG PET/MRI for Lymph Node Metastasis in Non-Small Cell Lung Cancer.


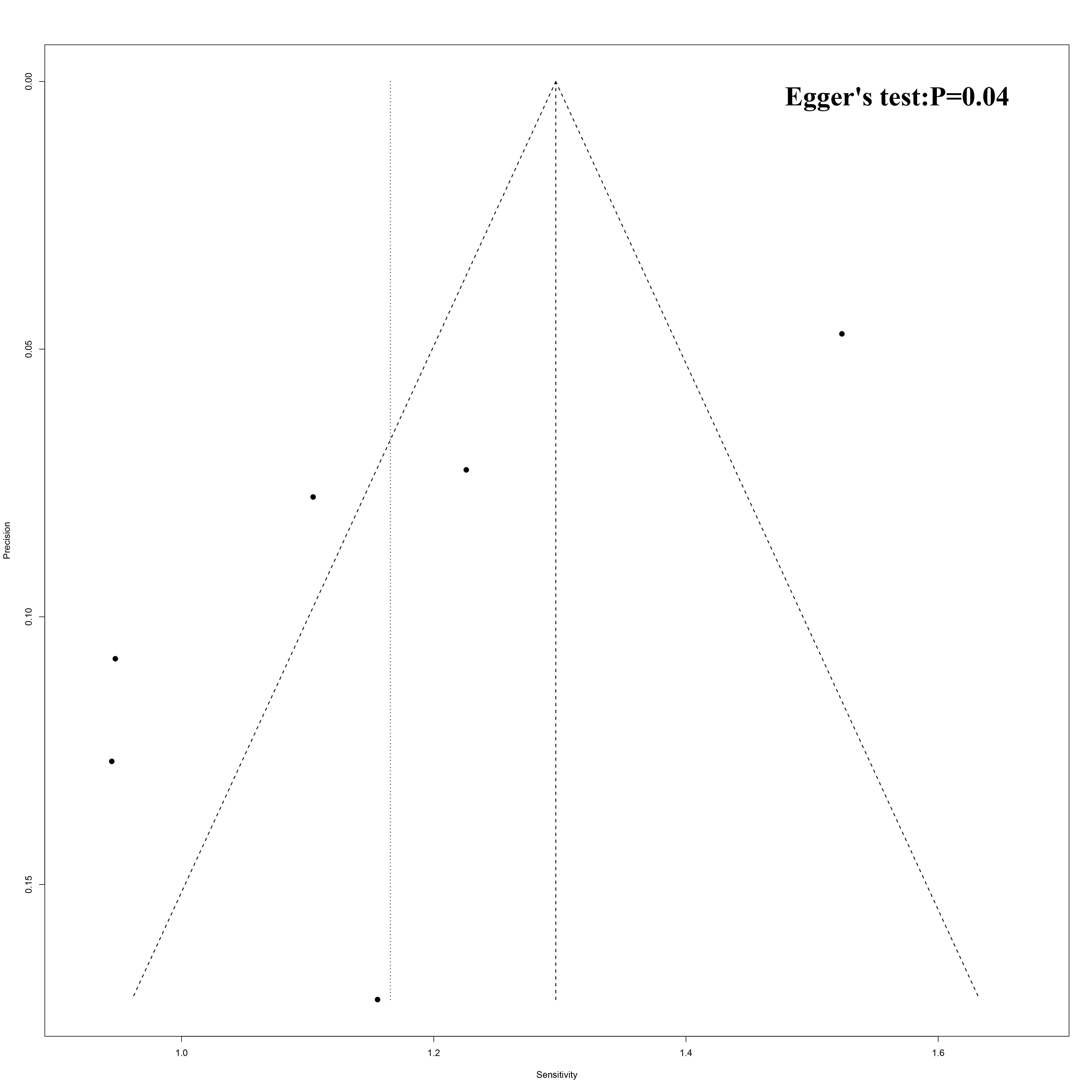


Supplementary Figure 7 Funnel Plot Assessing Publication Bias in Studies on Specificity of [18F]FDG PET/MRI for Lymph Node Metastasis in Non-Small Cell Lung Cancer.


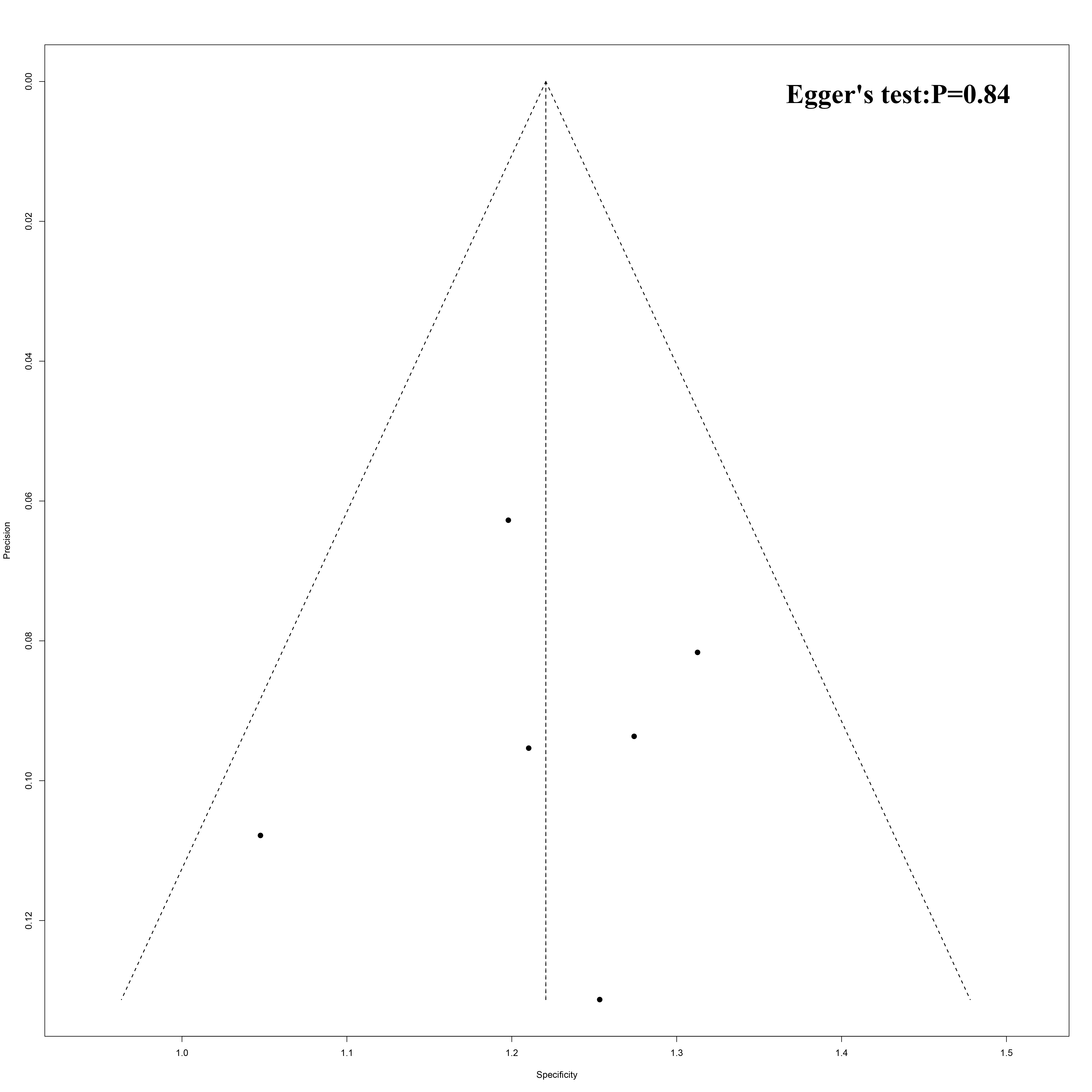

Supplement: Supplementary file 1 [file Data_Sheet_1.DOCX]
